# Family language policy retention across generations: childhood language policies, multilingualism experiences, and future language policies in multilingual emerging Canadian adults

**DOI:** 10.3389/fpsyg.2024.1394027

**Published:** 2024-09-10

**Authors:** Leah L. Pagé, Kimberly A. Noels

**Affiliations:** Department of Psychology, University of Alberta, Edmonton, AB, Canada

**Keywords:** family, language policy, home language, heritage language, retention, multilingualism, focus groups, Canada

## Abstract

**Introduction:**

Language policies in multilingual families refer to parents’ decisions, whether explicitly articulated or not, regarding which languages will be used in which contexts. However, because most studies that explore language allocation focus on families with young children, they do not address how family language policies impact the retention of a home language through to the next generation. The present study investigates an important perspective on this issue, specifically how emerging adults’ childhood experiences with their family language policy relate to the languages they currently use and plan to retain in the future.

**Methods:**

In all, 62 multilingual Canadian adults, aged between 17 and 29 years, participated in focus group interviews concerning their experience of language policies in their birth families, their current beliefs concerning language allocation and retention, and their plans about language policy in their future families.

**Results:**

The data revealed that not only are most participants interested in retaining their home language, thereby continuing to speak the language in their future families, but most are also open to incorporating additional languages into their policies.

**Discussion:**

The results provide insight into how to identify effective heritage language retention policies that transcend generations.

## Introduction

In many countries, ethnolinguistic diversity is argued to have important economic and civic advantages (Caraballo and Buitrago, 2019; Schroedler et al., 2023; Sokolovska, 2023). Given these benefits, maintaining minority languages within a society can be viewed as an important goal for a society. Language maintenance at the societal level is supported through language retention at the individual level (Yagmur and van de Vijver, 2022). A person’s early exposure to language impacts their use of language as an adult, making it important to understand how social norms at home and school influence children’s language beliefs and habits. These norms can either encourage or discourage children’s willingness to retain their home language (HL)^1^—a language “spoken or used in the home or community but which is not the majority language in the society” (Schalley and Eisenchlas, 2020, p. 2).

The social norms that affect children’s language allocation in the home are broadly framed as family language policies (FLPs) (Spolsky, 2004). Most of the research concerning FLPs and language allocation focuses on families with young children, typically under the age of 12 (e.g., Ballinger et al., 2022; Chatzidaki and Maligkoudi, 2013; Kaveh, 2020; Kaveh and Sandoval, 2020; King and Fogle, 2006; Lee, 2021; Li, 2006; Song, 2016; Surrain, 2021). While there are select studies that did address adolescents and adults (e.g., Fogle, 2013), most studies do not address how family language policies impact the retention of an HL to the next generation.

An important step in understanding whether and how HLs are passed down generationally is to understand how childhood ethnolinguistic experiences, including experiences with language policies, could impact young adults’ decisions about which language(s) to use in the future and whether those languages will be retained. During the transitional period between 17 and 29 years, emerging adults are often living more independently from their families of origin for the first time and figuring out who they are and how they want to live their lives. These decisions can include the role of language in their future family (i.e., future partner and children).

In this study, we aim to increase our understanding of HL retention by studying emerging adults’ attitudes toward childhood language policy retention and future language use. To that end, we consider how adults’ experiences with language policies in their homes and schools, and their beliefs about the opportunities, challenges and anxieties they experienced as multilingual speakers contribute to their intention to retain their languages and pass them on to their own children. Accordingly, this study builds upon the theoretical framework of FLPs and school language policies concerning HL retention.

### Home language retention and family language policy

Language retention refers to a person’s ability to uphold their HL while living in a society where the predominant language differs from their HL (Hyltenstam and Stroud, 1996). The decision to retain an HL could stem from either its functional role of obtaining services in that language (e.g., receiving help at the grocery store) or its symbolic roles, which include establishing a connection to cultural heritage (Kipp et al., 1995, as cited in Sussex, 1998). Whereas adults can make these decisions for themselves, younger children may rely on their parents and guardians to make these decisions for them.

Parents and guardians can implement family language policies (FLPs) to support the retention of an HL within their family. FLPs encompass a broad framework of “planning in relation to language use within the home among family members” (King et al., 2008, p. 907). This framework involves setting goals for language use, making decisions about which languages to use in various contexts, and developing strategies to encourage and support language allocation in different social situations.

Two significant situational domains in most children’s lives are the home and school settings. The domestic context is crucial to understanding language retention because the communication between a child and the primary caregiver is at the core of language transmission and retention (Bezcioglu-Goktolga and Yagmur, 2022). Many researchers have highlighted the importance of the home environment in the development of language attitudes as well as language retention (e.g., Bezcioglu-Goktolga and Yagmur, 2022; Hollebeke et al., 2022; Li, 2006). Hollebeke et al. (2022) argue that “intergenerational transmission makes families the cornerstone of heritage language maintenance” (Hollebeke et al., 2022, p. 3) because parental use and preservation of an HL provide children with essential language exposure in the home environment.

When a parent’s HL differs from the societal language (SL), that is, the dominant language in a given area (e.g., English in Western Canada; Estonian in Estonia; Mandarin Chinese in Taiwan), the acquisition of an SL may interfere with the mastery of an HL (Hollebeke et al., 2022). Because of this linguistic dominance, some parents may decide to enforce stricter rules involving HL use within the household to prevent childhood language loss. Continuous exposure to an HL at home plays the most pivotal role in deciding whether or not the HL will be preserved or neglected over generations (Park and Sarkar, 2007). Above all, when an HL is recognized as a core value by a child’s parents and presumably, the child, the feasibility of language retention is increased (Bezcioglu-Goktolga and Yagmur, 2022). Investigating language values instilled in the home environment during childhood will better help us understand long-term language retention practices.

Once children are old enough to enter the educational system, FLPs can be influenced by language rules at school. School language policies refer to plans implemented by school boards to support students’ language acquisition and development, providing opportunities to improve pupils’ literacy and language practices (Vanbuel and den Branden, 2021). According to Curdt-Christiansen (2022), families and schools must collaborate in creating a linguistic environment that fosters the development of both students’ HL and SL. For instance, Sandel (2003) investigated the impact of language policies enforced in Taiwanese schools and subsequent language attitudes and speaking practices at home. After decades of students being prohibited from learning local languages or dialects at school, the policy was reversed in 2001 allowing these languages to be included in school curriculums (Sandel, 2003). The data show that for older generations, speaking Tai-gi is seen as a shameful thing because it labels a speaker as being uneducated. In comparison, younger generations were formally taught in both Mandarin and Tai-gi at school and have stressed the importance of speaking both dialects fluently (Sandel, 2003). As evidenced by Sandel (2003), FLPs transcend domestic boundaries and are subject to rules in educational domains.

Similar to the Taiwanese context, school language policies are an influential factor for future language use in Canada as well. Slavkov (2017) investigated the effects of language policy at home and school language choice on the subsequent multilingualism of children living in Ontario, a Canadian province with English as the SL. The languages participants spoke at home were a combination of exclusively an HL, mostly an HL, mostly the SL, and exclusively the SL. The data show that the language of communication between siblings, a child’s minority language literacy, and the language spoken between parents were the most influential factors in a child’s language use (Slavkov, 2017). In terms of language in an educational context, minority language programs such as Francophone schools, where instruction is only provided in French, and immersion programs, a technique where the SL and HL are used in instruction to varying degrees as students mature through the program, were shown to be positively associated with long-term multilingualism (Slavkov, 2017). Therefore, we can conclude that the interplay between appropriate FLPs and schooling in a minority language can increase the likelihood of children remaining multilingual and retaining their home languages.

Throughout Canada, the linguistic climate is quite diverse. French is spoken by 84.1% of Québec’s population (Statistics Canada, 2024a). With this specific ethnolinguistic context in mind, Ballinger et al. (2022) investigated the language beliefs and practices of first-time parents raising multilingual children in Québec, along with their thoughts on societal language policies in conjunction with FLPs. The results demonstrate a “complex co-existence” (Ballinger et al., 2022, p. 614) of family and official language policy. Participants stressed the importance of instilling a strong French language foundation in their children through formal French education, considering the language as a form of “cultural capital” (Ballinger et al., 2022, p. 623). Even if French was not spoken at home, parents wanted to indemnify this linguistic gap by enrolling their children in French schools, despite being eligible for English programs (Ballinger et al., 2022). Subsequently, when addressing FLP retention in the current study, it is crucial to confront the interconnected and influential variables of school and societal language policies and policies enforced at home.

### Explicit versus implicit FLPs

Within educational contexts, language policies are often formally articulated; in family contexts, more variety is often present. In some families, parents provide explicitly stated expectations for the language to be used with parents, siblings, and extended family members, along with clear consequences if the expectations are not met. Some researchers, however, have raised concerns regarding the practicality of intentional language rules within the home. Palviainen and Boyd (2013) argue that, although FLPs are planned, conscious, and motivated in theory, in practice, FLPs are often implicit, reflecting often unconscious and organic patterns of language use within the family. These “unstated but usually seen practices” (Li et al., 2022, p. 3375) are referred to as implicit FLPs. These implicit belief systems can be quite diverse, reflecting broader ideological stances regarding the appropriateness of languages in different social domains (Lanza, 2007).

There is debate regarding which type of policy is more effective in instilling long-term multilingualism in young children (King et al., 2008; Palviainen and Boyd, 2013). Some research shows that families who embrace more deliberate and HL-oriented rules at home are more likely to retain their HL because the children’s HL exposure is maximized. For instance, Hollebeke et al. (2022) researched indicators of parental HL retention efforts in multilingual families within the Flemish community of Belgium. Their analyses revealed a positive correlation between explicit family policies and HL retention efforts. This finding aligns with those of King et al. (2008) who showed that FLPs should be overt, definite, and planned to instill bilingualism/multilingualism within a child.

### Language retention into the next generation

FLPs, whether explicit or implicit, can provide a road map for how language is to be used within a family. They do not guarantee, however, that offspring will hold the same linguistic attitudes and values as their parents concerning the successive retention of the language or the FLP they grew up with. Bezcioglu-Goktolga and Yagmur (2022) investigated the differences in language attitudes between first and second-generation Turkish parents living in the Netherlands. Although both generations displayed a preference for bilingualism, second-generation participants spoke Turkish less than their first-generation counterparts. In fact, they used Dutch more commonly than Turkish in daily conversation at home and engaged in language management activities less often than first-generation parents. These findings highlight the possibility of discrepancies between linguistic values and the execution of language management across immigration generations.

In addition to policy around the HL, decisions need to be made about the use of SLs. Bilingual and multilingual offspring’s beliefs are shaped by their awareness of language ideologies regarding the utility and value of languages. In a study investigating the relationship between English-only school language policies and FLPs in eight immigrant families to the United States, Kaveh and Sandoval (2020) found that similar to previous studies, for second-generation immigrant children, English was an indicator of academic achievement as well as a tool for survival and belonging within their societal context. Not only can language retention function as a “link between the generations and cultural values of the ethnolinguistic group” (Schwartz, 2010, p. 175), but this finding demonstrates the functionality of the language. Not all multilingual speakers consider all languages as a vital part of their identity; some function solely as tools for communication.

### Multilingual experiences: opportunities, challenges, and anxieties

Family and school language policies can reasonably be expected to impact people’s decisions about language retention; experiences around multilingualism can also have important implications for people’s beliefs and values regarding language retention. Haukås et al. (2022) investigated Norwegian student beliefs about the potential benefits of speaking multiple languages. Interestingly, school language policies and academic linguistic requirements were seen to have less effect on participants’ language beliefs compared to “extramural experiences” (Haukås et al., 2022, p. 10). For example, participants with migration backgrounds, with friends with a home language other than Norwegian, and who have had experiences living abroad had significantly more positive perceptions of multilingualism compared to participants lacking such experiences. These advantages include the development of perspective-taking skills, the ease of learning additional languages, and improved language awareness (Haukås et al., 2022).

Although a multilingual upbringing can have many advantages, it can also have social and personal challenges. Newcomer (2020) conducted a study in the “particularly restrictive context of Arizona” (p. 194), where bilingual education had been prohibited since 2000, despite research stressing the effectiveness of additional languages taught in schools. The study touched upon bilingual and bicultural high school students’ experiences of microaggressions, such as the mispronunciation of names. One participant expressed “I was considering changing my name because I thought people would have an easier time saying it. That is how stressed I was with the whole name situation” (Newcomer, 2020, p. 201). Other consequences Newcomer (2020) identified from the English-only policies at school include cultural loss, academic difficulty, diminished opportunities for success, and family disconnect.

Implementing FLPs that support bilingual language practices may pose some challenges to parents living in a monolingual linguistic context. For example, Seo (2022) conducted several semi-structured interviews to examine the challenges parents face in implementing bilingual parenting in the context of Korea. The study identified two primary challenges: a parent’s lack of English proficiency and differing perspectives between spouses regarding their children’s language development. Specifically, parents resisted implementing an English-only rule at home due to varying family members’ views on language practices (Seo, 2022).

Challenges with multilingualism can give rise to negative emotions, including language anxiety, or the apprehension a language user experiences when expected to perform in a particular language (Sevinç and Dewaele, 2018). Although one might expect to feel language anxiety when communicating in an SL, this form of anxiety also can be present when communicating in an HL in domestic contexts. According to Hollebeke et al. (2022), if parents view multilingualism as culturally, economically, and socio-emotionally beneficial, they are more inclined to consciously endorse HL development and a multilingual mindset. Conversely, when multilingualism is negatively perceived due to aggressive monolingualism and a single-language mindset, often combined with the expectation of perfect fluency in both languages in multilingual individuals, this restrictive mindset can hinder their healthy engagement with language opportunities, potentially leading to negative experiences like stress and anxiety (Sevinç, 2022).

### The Canadian context

The societal value placed on language and multilingualism in Canada, the context of the current study, shapes Canadians’ perceptions of the importance of language. This country is known for its official bilingualism, multiculturalism policy, history of promoting home languages and cultures (see Noels and Berry, 2016, for an overview), and, more recently, its dedication to the preservation, promotion, and revitalization of Indigenous languages (Canadian Heritage, 2024). Under the Official Languages Act of 1969, English and French were proclaimed Canada’s official languages. This Act not only inaugurated official bilingualism in legislative bodies but also gave English and French official status in institutions and organizations under federal jurisdiction, such as postal services and air transportation (University of Ottawa, 2024).

Aside from the official languages, Canada is becoming increasingly multilingual. According to Statistics Canada (2022a), 41.2% of Canadians were able to converse in more than one language in 2021, a significant increase from 39% in 2016. And according to the 2021 Canada Census, one in five Canadian households (21%) was multilingual in 2021 (Statistics Canada, 2023a). This increase is likely due to an increase in immigration. Almost a quarter of the Canadian population (23%) were landed immigrants in 2021, a proportion that is the highest in Canada’s history since Confederation as well as the highest proportion among all G7 countries (Statistics Canada, 2022b). Canada has “a rich linguistic diversity” (Statistics Canada, 2022a) and because of this richness, language retention is a very prominent and necessary topic to research.

Canada is a large country, and regions therein can differ in the number and diversity of languages spoken. Like Canada more generally, the provincial context for this study is ethnolinguistically diverse. Apart from the federal official languages of English and French, other languages that are commonly spoken in Alberta are Chinese, Filipino, and South Asian languages, in addition to over 50 other languages (Statistics Canada, 2023b). Although the province of Alberta has declared English its official language, Francophones, who comprise less than 2% of the provincial population (Auclair et al., 2023), have the right to education and federal services in French. Until 2022, Alberta students were required to learn a language other than English (or French, if enrolled in the French system) between grades 4 and 9. There is no requirement that non-Francophones learn French in public schools; nonetheless, French is the most commonly offered and studied language, whether through second language courses and/or French immersion programs (Alberta Ministry of Arts, Culture and Status of Women, 2024). The public education system also includes eight other bilingual programs in international languages including Arabic, Chinese, German, Italian, Japanese, Punjabi, Spanish, and Ukrainian, as well as language and culture courses in these languages. Programs and courses for additional languages can be created where numbers are justified. Many non-official language communities also organize community language courses separate from the public school system (International and Heritage Languages Association, 2024). In sum, Canada, and Alberta specifically, is ethnolinguistically diverse, and residents and citizens have multiple and complex opportunities, across formal and informal situational contexts, to learn and use English, French, their HL, and other languages throughout their lifespan.

### The current study

Within this multilingual context, the present study examines multilingual emerging adults’ experience of multilingualism and family language policies, using the following research questions to guide our study:

1. What are adult participants’ childhood experiences concerning family and school language policies?
2. How are their experiences of multilingualism related to the articulation of their own attitudes and beliefs about language policies?
3. Do they wish to retain their HL, and if so, what do they imagine as their future FLP?

## Methods

Focus group interviews were chosen for the method of data collection because this study’s purpose was to explore a wide range of experiences with languages, FLPs, and future intentions. Like other qualitative methods, focus groups allow a greater degree of in-depth exploration of focal topics than do numeric rating scales and other questionnaire survey instruments. In contrast to 1:1 interviews, the facilitated discussion in focus groups requires participants to clearly articulate their experiences for a diverse audience and allows participants to build off of or counter other people’s ideas (Gammie et al., 2017). These features were expected to effectively elicit a wide range of insightful perspectives while remaining time and resource-efficient (Gammie et al., 2017).

### Participants

Because this study concerned multilingual emerging Canadian adults, we restricted the inclusion criteria for participants to people between the ages of 17 and 29 who were Canadian citizens who spoke more than one language during childhood and who were not currently parents. Given that this study was designed to elicit a wide range of experiences and opinions, and because it is common for Canadians to be exposed to English, French, and many other languages throughout their lifetimes, whether in Canada or other countries, we included speakers of any languages, whether official or non-official languages. Due to this linguistic diversity, many undergraduate students at the Western Canadian University were eligible to participate. However, within the timeframe allotted to collect the data, we had a limited number of students who signed up for our study.

In all, we recruited 62 participants (39 female and 23 male) between the ages of 17 and 29 (M = 19.26 years; SD = 1.37%) from the psychology research participation pool at a Western Canadian university. With this research participation pool program, students enrolled in an introductory psychology course receive course credit by signing up for psychology studies. One participant was omitted from the analyses because they did not meet the study’s inclusion criteria for age as they were over 30 years old and had children. Approximately half of the participants indicated that they immigrated to Canada (*n* = 32), a quarter indicated that they were born in Canada to an immigrant family (*n* = 13), and one participant specified they are a third-generation Canadian (16 did not respond). Most participants (70.5%) spoke two languages (English and a language other than English (LOTE)), almost a quarter (23%) spoke 3 languages (English and two LOTEs), and the remainder spoke 4 (3.3%; English and three LOTEs) or 5 (3.3%; English and four LOTEs) languages. In all, 29 languages other than English were represented, including Hindi (*n* = 13), Urdu (*n* = 11), Arabic (*n* = 8), French (*n* = 8), and Punjabi (*n* = 8). With regards to French, no participant learned French as a familial language; it was primarily learned through the education system. A complete breakdown of the languages spoken can be found in Supplementary Appendix 1.

### Procedure and materials

The data were collected through online focus group interviews over Zoom that were recorded using Zoom’s Record to the Cloud function and transcribed using Zoom’s Live Captioning function. The focus group interviews were conducted in English and because we recruited university students studying at an English university in Western Canada, which has both written and spoken English proficiency requirements, we did not test the participants’ English language proficiency.

Twelve 60-min focus group interviews were conducted with the number of participants ranging from two to eight students in each session. The sessions were conducted in English and the discussions revolved around topics such as (1) language use at home and school and the relative implicitness or explicitness of FLPs; (2) opportunities and challenges related to bilingualism/multilingualism, events that caused changes in language use, and anxieties regarding multilingualism; and (3) thoughts concerning future language use and FLPs. Before the interview questions were posed, participants were given the definitions of language allocation and family language policies. The interview questions can be found in Supplementary Appendix 2.

### Data preparation and analysis

After the recordings were transcribed and anonymized, we reviewed the recordings to ensure the transcriptions were accurate. Minor modifications were made (e.g., removing filler words such as “umm” and “like”) to make the transcriptions clearer and more concise.

The anonymization process focused on removing identifiable information such as names rather than dissociating responses across questions. We coded the individual responses to each interview question, which we could then link through identifiers assigned to each participant (e.g., interview number seven, participant number five). Therefore, we could make connections across different interview questions for the same participant without revealing their identity.

The results were organized based on the different focus group interview questions. Using NVivo, the transcribed interviews were coded into themes, separately for each interview question (see Supplementary Appendix 2; Table 1). The themes were developed through a process of constant comparative analysis (Glaser, 1965; Boeije, 2002), which requires the analyst to “compare [each incident] with the previous incidents in the same and different groups coded in the same category” (p. 106), creating categories until the analyst is satisfied that no more categories emerge from the data. To ensure the trustworthiness of the findings, we adopted the evaluative criteria as outlined by Lincoln and Guba (1985; see also Cohen and Crabtree, 2006) to the extent possible with focus group interviews. Multiple quotations were provided for each coding category (i.e., thick descriptions) and particular attention was directed to finding cases that deviated from the general trends (i.e., negative case analysis). The first author completely reviewed and coded the transcripts, and then recoded a subset 1 week later. The second author independently reviewed the transcripts in reference to the coding system to confirm the first author’s decisions (i.e., triangulation; audit trail), and any discrepancies were resolved through discussion (i.e., reflexivity). Some techniques were not possible due to the regulations of the research participation pool and/or the research ethics board; specifically, because participants cannot be contacted after their commitment to the project has been fulfilled, we could not extend engagement after the interview session and/or check our interpretations with the participants (i.e., prolonged engagement, member-checking).

**Table 1:** Overview of the themes derived from each question and corresponding research questions addressed by each interview question.

Question posed	Table number	Number of themes and subthemes	Corresponding research question addressed
What language policies were you exposed to at home?	2	3 themes and 3 sub-themes	RQ1
What language policies were you exposed to at school?	3	2 themes	RQ1
What are some of the challenges you faced because you can speak more than one language?	4	4 themes and 15 subthemes	RQ2
What are some of the opportunities you had because you can speak more than one language?	5	4 themes and 10 subthemes	RQ2
Were there any events in your life that caused a change in your language use and if so, what were they?	6	3 themes and 8 subthemes	RQ2
Have you ever experienced anxiety when speaking one of your languages and if so, what was the reason for this anxiety?	7	8 themes	RQ2
How important is it for your significant other to speak all your languages?	8	5 themes	RQ3
Are you interested in retaining your language policies?	9	3 themes	RQ3

## Results

Table 1 summarizes the themes derived from each interview question and their relation to the research question(s). For each theme and sub-theme, we counted the frequency of how many participants referred to that (sub-) theme to make our analyses show regularities (and some peculiarities), and point to possible transferability to other settings (Maxwell, 2010). The (sub-) themes are described below in the order in which the question was asked in the interview.

### Family language policy

When asked about the nature and structure of the FLPs that they recalled from their childhood, 51 different responses were given by the participants (see Table 2). A total of 20 participants stated that they were exposed to an explicit FLP, such that they were given strict rules regarding language use. Within the theme *explicit policy*, three sub-themes emerged. Nine participants were explicitly told by their parents/guardians to speak their HL.

**Table 2:** Summary of themes for family language policy.

Theme	Subtheme	Number of responses	Number of participants	Example
Explicit
	Explicitly told to speak their HL	15	9	“So at home, my parents very heavily encouraged us to speak in Urdu, so that we remember the language, and are able to speak it to our elders back home in Pakistan.”
	More relaxed over time	3	3	“I used to be told a lot more when I was younger to speak Spanish and French because my parents were scared that I was gonna lose them when I was younger. And then as I got older, they were like, okay, she can speak them. They started to put less pressure on me.”
	Explicitly told to speak English	2	2	“I was told by my parents to speak exclusively English. I think this was when I was around seven or eight, and only Tagalog at home. This influenced the language I use today.”
Implicit		31	25	“My parents never enforced it on me, but I was interested in the language of my heritage.”
Both implicit and explicit		1	1	“For me, it was a bit of the both.”

In addition, three participants expressed a policy experience that gradually became less strict over time, as noted by one participant:

“For me, I think, earlier on during elementary, my parents did want me to speak mainly Korean at home, but as I got older, I think that they were more relaxed with that rule because they knew that I was already sufficient enough in Korean.”

For many families, language rules are not static. Instead, they are dynamic, changing with the development of the child.

The third sub-theme was parents urging their children to *speak English* (*n* = 2). For example, one participant stated: “My parents did not know much English, so they encouraged me to speak English to them, and then eventually they learned through me.”

Another 31 participants who labeled their childhood FLPs as implicit. They were never told explicitly which language they had to speak, but rather allocated language based on environmental clues and the languages that other people were speaking. For example, one participant stated: “It was just whatever was appropriate based on previous contextual knowledge of what the person spoke.” Lastly, one participant mentioned that the policy they were exposed to at home was a combination of explicit and implicit implementation.

### School language policy

The participants’ experiences with languages in schools were diverse, and often complicated by histories of migration and the range of opportunities for language education in Canada and elsewhere. Some participants began to learn English before migrating to Canada (e.g., through SL courses or British schools, etc.), and/or learned other national languages depending on their place of residence before arriving in Canada (e.g., Italian after landing in Italy after leaving their home country), and/or learned a familial language while sojourning in their country of origin (after migrating to Canada). While living in Canada, some took English SL courses, some participated in bilingual programs in their HL, and some studied other languages through bilingual programs or SL courses. Many reported that they studied French at some point in their education, either through immersion or in an SL course, sometimes both at different times. At least two respondents spent their first years in Canada in the Province of Québec, where they were educated in French. It is noteworthy that some students reported that although they had the opportunity to study a language from their country of origin, they were not necessarily able to study their home language (e.g., a bilingual Tagalog program is offered, but not other Filipino languages).

With regard to school language policies, most students noted that the expectation was that they would speak the language that they were studying in the language course. A total of 27 participants stated that language classes were mandatory and once the classes became optional, they did not have the motivation to continue pursuing them (see Table 3). The other 26 participants indicated that even though additional language classes were mandatory for a couple of years, they continued to take the language courses once they became optional. For example: “French was mandatory until grade 10, and then I took it as an options class for 2 years.” In addition, some participants are actively engaging in language learning endeavors in their university careers: “I’m actually finding myself wanting to take more language classes. Even now in university (Table 4).”

**Table 3:** Summary of themes for school policy type.

Theme	Number of responses	Number of participants	Example
Mandatory	38	27	“I see the benefit to learning languages now, but growing up I did not try as hard at school because I felt forced to do it.”
Voluntary	39	26	“I also did pursue [French] in high school, even though it was optional back then.”

**Table 4:** Summary of themes for the question “What are some of the challenges you faced because you can speak more than one language?”.

Theme	Subtheme	Number of responses	Number of participants	Example
Language skills and awareness
	Cannot break it down	1	1	“When it comes to a language that you have been raised in, you cannot break it down anymore. How much do you really understand the language? If you cannot even like, break it down into its simplest parts.”
	Confusing or mixing languages	18	12	“I’ve had situations where I’ve been talking to a teacher and I’ve accidentally said a number or a word in Punjabi, Hindi, or Urdu.”
	Difficulty expressing	6	6	“I cannot articulate everything that I want to say in my thoughts, or I do not know if it is gonna make sense.”
	Loss of language	5	4	“Arabic does not come as naturally to me anymore. So, it takes a while to think of the right phrase or the translation to an English word. I do not even feel confident putting it on my resume because of how anxious I am.”
	No similarities	2	2	“I found that it was extremely difficult because I do not think there are very many similarities between the two languages. It just felt like I was trying to battle learning English and Ukrainian.”
	Not sure what language to use	1	1	“I work at a grocery store and I come across lots of people that speak Punjabi. But I always say that it’s a challenge for me because I never know whether they want me to speak to them in English or in Punjabi.”
Restricted opportunities
	Isolation	3	1	“It was quite isolating, just being stuck with the same group of 21 kids for seven years. And then all of a sudden, you get to middle school. And now you are expected to make new friends and be with people that you have not been with for the last forever. So it’s definitely quite difficult.”
	No one speaks the language	1	1	“The hardest part for me is finding people I can converse with in my language. Because moving here, I can count on one hand how many people I’ve met that can speak [Bisaya] so it’s really hard to keep.
Social judgment
	Discrimination	3	3	“Being called certain words, or being called out for speaking Punjabi in order to translate certain things with like my classmates, in order to understand and comprehend better the English language. I think those would be some of the challenges that I faced with the transition to a whole new different country.”
	Harsh language	1	1	“[Pashto] sounds like a really angry language. So sometimes I’ll be talking to someone, and then people say “Are you angry?” I’m not. That’s just how it sounds because the words are spikier.”
	Judgmental looks	2	2	“The main negative thing would be the fact that some people would give you a side eye or they’d be kind of judgmental about whether we are speaking of them. Or maybe we just like trying to exclude them from some conversation.”
	Mispronounce name	4	4	“For me, people kept mispronouncing my name. I kind of adopted that for a while, until I took pride in its proper pronunciation.”
	Pressure or expectations	2	2	“There’s definitely a pressure to [retain] both languages skillfully, especially if you are not exposed to both languages at the same level of intensity. Then [retaining] one of your languages can definitely be a struggle.”
Other
	Having to learn a language	2	2	“I kind of struggle with learning languages in general, so mine is kind of the opposite of what most people have said.”
	No challenges	7	7	“I do not really think I’ve ever experienced any downside to speaking another language.”

### Challenges with multilingualism

When we asked our participants about the challenges associated with multilingualism, 59 responses were given. The most common response was confusing or mixing languages (*n* = 18), specifically regarding the difficulty in allocating language. For example:

“So sometimes, being multilingual gives you a challenge that you speak the wrong language to the wrong person. For example, sometimes I will say some phrases in Urdu to a person who just completely speaks in English.”

It is important to discuss the distinction between two related themes in Table 5: *loss of language* and *difficulty expressing*. While it is true that having difficulty expressing oneself in an HL can stem from gradual disuse or loss of language, not all people who have difficulty expressing themselves in one of their languages are losing that language. According to Baker and Wright (2017), there are four dimensions of language skills—listening, reading, speaking, and writing—that are measured along two dimensions: receptive/productive skills and oracy/literacy. The idealized perception of a balanced bilingual, a person who is equally proficient in the four language skill dimensions in all their languages, is quite rare. Therefore, an individual’s proficiency in multiple languages is “multidimensional and will tend to evade simple categorization” (Baker and Wright, 2017, p. 7). A person facing difficulty speaking their HL can still be considered bilingual if they display more receptive language skills such as listening and reading; this fact is exemplified in the distinction between the two themes of *loss of language* and *difficulty expressing*.

**Table 5:** Summary of themes for the question “What are some of the opportunities you had because you can speak more than one language?”.

Theme	Subtheme	Number of responses	Number of participants	Example
Cultural engagement
	Holidays or festivals	3	1	“I feel like being Ukrainian and knowing Ukrainian has opened me up to a lot of cultural stuff, which is quite fun, not only with, say, dancing or the food or just community stuff. It’s quite nice to be a part of it, the holidays and different things.”
	Religion	3	3	“Another really important thing in my life is my religion, Islam. Our holy Book is in Arabic and even translating it to English, the words aren’t perfectly aligned so it does not give you the same experience reading it. If I had not known Arabic, it would be very difficult for me to be Muslim.”
Instrumental opportunities
	Job opportunities	5	5	“I hope to work on an international level someday. So, this is definitely an advantage.”
	Travel	6	6	“One of the best advantages is just how much easier it is to travel around, just because you know more languages.”
	University	3	3	“Speaking good English allowed me to take the [International Baccalaureate] program and that program was probably a big reason why I got accepted here into computer science.”
Social connection
	Anonymity	6	6	“I think, for me personally, I like speaking Spanish so that other people cannot understand what I’m saying.”		Connection and communication	36	31	“Being able to speak Punjabi is really important to me because it’s the only way I can communicate with my grandparents because they do not know English.”
	Translating for other people	2	2	“So, one thing that I do that I think is really cool is translating for other people. […] I just think it was really cool to help others who did not understand the language and to translate for them. I thought it was really helpful.”
Other
	Language similarities	10	8	“Arabic and French have a lot of similarities, so I feel like you know, being fluent in Arabic, would also help me master French.”
	No opportunities	2	2	“But in my day-to-day life, I do not think there are many opportunities given due to the fact I speak Russian. Cuz I’m in an English environment. The fact that I speak Russian has very little effect on my life.”

Out of the 59 responses, seven participants did not associate speaking multiple languages with any challenges. These seven participants only associated speaking multiple languages with advantages. For example: “It was mostly like good experiences. So yeah, I do not think I’ve had any challenges.”

### Opportunities with multilingualism

When we asked our participants about the advantages associated with bilingualism/multilingualism, 83 answers were given which were divided into four themes. The most common benefit listed was being able to connect and communicate with a wider demographic of people. For example, one participant stressed the importance of connecting with people who spoke the same HL:

“I feel like you can build a closer relationship if some of your backgrounds are similar, and that’s how it was for me and my friends like a lot of my friends can speak Arabic.”

Two participants emerged as negative cases (i.e., representations of the uncommon cases that deviate from the general trend; Cohen and Crabtree, 2006); that is, neither associated speaking multiple languages with any benefits (in Table 5, this is labeled under the theme *no opportunities*). In both cases, the lack of opportunity to use the language regularly precluded any benefits from accumulating. As one participant stated:

“To be honest in Canada, I think the Polish language is fairly useless. Mainly because the only time I have ever used the Polish language is whenever I go into a Polish community. […] Other than that, I have never used Polish for just regular use.”

### Changes in language use

The event most participants listed as a catalyst for the change in their language use was moving to another country (*n* = 37). For most participants born or raised in a country other than Canada, moving to Canada caused an increase in English use and a decrease in HL use. When participants went back home to their country of origin, the result was an increase in the use of their HLs. As one participant stated: “Any time that I’m back home, I think my Urdu always significantly improves.” In many of the cases where respondents commented on changes in language use, an increase in speaking English was accompanied by a simultaneous decrease in speaking an HL.

Two types of negative cases to this trend were identified. The first negative case is labeled in Table 6 with the theme *increase*. Despite moving countries or changing programs in school, two participants maintained that there was an increase in HL use. One participant stated: “My language use, I feel like, increased. I think it was just because of time, I just got better at using the language and understanding it as I got older.”

**Table 6:** Summary of themes for the question “Were there any events in your life that caused a change in your language use and if so, what were they?”.

Theme	Subtheme	Number of responses	Number of participants	Example
Education
	Changing programs	1	1	“The separation between when we actually finally got split up as a class was different because we still had the Ukrainian program in the Ukrainian classes. We just were not all together all the time. I find that once we kind of got separated, we all went to the real world, almost, you know, not just this world we have made. That was a big thing because I feel just less forced to speak it.”
	Changing schools	6	6	“My way of speaking to my parents has changed a lot since I moved to university, as before it was mainly Hindi, but now I speak English as well with them.”
	Started school	8	6	“I think starting school because from what I remember when I was super young, I do not think I spoke a lot of English at home. But then, when I went to school, I was surrounded by English more. So, I kind of just started speaking it at home as well.”
Rare case
	Increase	2	2	“I think it’s increased, like talking more in Farsi. Because as I grew older, I realized, maybe I want to teach my kids it as well. So, I should be fluent in it.”
	No change	5	4	“I think, for me, because I immigrated at such a young age. There wasn’t any event that changed the way I use language. I already did not know much Russian and I do not know much English either, so I was kind of starting from the bottom with both languages growing up.
Other
	Loss of connection	2	1	“I do not really practice much now, because all the Ukrainian speakers in my family have now passed so I do not really have anyone to practice that language with.”
	Moving countries	43	37	“My fluency in Arabic definitely decreased over the years. And yeah, I think that was the big thing for me, like moving to Canada.”
	Parent enforced a new policy	2	2	“I spoke Tagalog primarily until my parents told me to use English exclusively. Took a few years after that. But then I switched to English, and my skill with Tagalog faded. I tried to speak it these days so it does not get completely forgotten.”

The second negative case is labeled in Table 6 with the theme *no change*. Similar to the theme *increase*, five participants argued that despite a major life event such as a move, their language use remained the same. One participant said: “Nope. I still speak Spanish with my family today. That has not changed at all.” One possible explanation for this lack of change in language use is the age at which participants immigrated to Canada. If immigration occurred at a younger age, it is less likely to have an effect since a child is learning the SL and HL simultaneously. Another possible explanation for these negative cases is the participants’ values and motivational orientations; one participant’s HL use increased because they would like to pass it on to their children in the future.

### Experiences of language anxiety

When asked whether the participants had experienced any instances of language anxiety, most indicated they had (*n* = 49 of language anxiety; *n* = 10 of no language anxiety; see Table 7). Consistent with Sevinç’s (2022) observation that one can experience anxiety with using either or both the HL or the SL, both types of anxiety were reported. For both languages, the most common reason for experiencing anxiety was a fear of making mistakes. Here is one example of a participant expressing an SL anxiety experience:

**Table 7:** Summary of themes for the question “Have you ever experienced anxiety when speaking one of your languages and if so, what was the reason for this anxiety?”.

Theme	Number of responses	Number of participants	Example
Difficulty expressing	12	10	“When I speak to someone that is better at the language, typically older than me. It’s a little bit nerve-racking because they will comment on your ability to speak back.”	Fear of making mistakes	14	13	“I’m scared of making mistakes and stuff like that, especially with my family.”
Fear of not being understood	3	2	“My grammar might not be good enough, and I’m always worried about it. Will they understand me? Will they be okay with it?”
Fear of not fitting in	3	3	“Sometimes I feel a little bit rejected or embarrassed when talking to my friends because I have a really thick Italian accent for some words. So, I kind of just feel embarrassed, like I do not blend in in most situations.”
Mixing dialects or accents	9	9	“It would make me nervous sometimes when there is a group of different nationalities, and then I would switch between the dialects. It felt weird to me. So now, mainly when I speak Arabic, it’s a mixture of a lot of dialects. So, I think that’s where some anxiety comes from. Where I do not have one clear, coherent dialect that I speak, but rather it’s a mixture of many.”
No anxiety	10	10	“It’s kind of the opposite for me in the sense that, I do not feel anxious speaking it to my parents and my family, because I’m not really thinking about it at the time.”
Not fluent	9	9	“I definitely feel anxiety from being forced to speak Mandarin given I’m not fluent enough.”
This meeting	2	2	“I often find myself before, for example, even this meeting, or even before giving presentations, I do find myself anxious that I need to communicate.”

“I was not that comfortable with speaking in English all the time and communicating with native speakers was hard. When I came to Canada it was kind of difficult for me, because I thought that I was sounding kind of off, and people were going to judge me or something. So, I just tried to speak less in the beginning.”

Conversely, here is an example of a participant expressing an HL anxiety experience:

“So sometimes when I tell people I speak Spanish, and there’s like an adult that also speaks Spanish, I get nervous because I don’t know how to be because since I only ever spoke Spanish to my family, I don’t know how to be formal and informal. So I just get really nervous because I’m afraid that I’ll be disrespectful when talking to them.”

In both cases of SL and HL anxiety from the quotes above, the participants expressed the experiences tended to occur in the presence of older generations or others who were more fluent than themselves.

A total of 10 participants emerged as negative cases during data analysis. Similar to the participants who did not associate bilingualism/multilingualism with any disadvantages, these participants recalled no instances of language anxiety. For example, one participant said.

“I for one haven’t experienced this. If anything, I’m actually proud to be able to speak different languages and try to show off how well I can actually speak.”

### Future language use and policies

After asking our participants about their experiences as multilingual speakers, we then prompted them to think about the future in terms of how language will be used in their own homes. We first asked our participants if it was important for them that their future partner spoke, understood, or simply respected all the languages that they spoke. The responses (*n* = 54; see Table 8) were quite diverse, with 14 participants articulating that they would prefer if their partner was already fluent in their HL, either already being able to speak or understand the language. This preference is not only related to communication with family members but also related to FLP retention (as seen in the example in Table 8).

**Table 8:** Summary of themes for the question “How important is it for your significant other to speak all your languages?”.

Theme	Number of responses	Number of participants	Example
Respect	18	13	“If I want my partner to respect the languages I speak and how I want them to be integrated into my life, I feel like I have to do the same for my partner.”	Open to learning	18	13	“I feel like if you are gonna be a part of my family, you are gonna have to at least try to learn my language.”
Speak/understand	14	14	“I think for me it’s important that my future kids speak Arabic. So, it would be great if my significant other also spoke Arabic.”
One common language	11	10	“As long as we have a common language that we are able to understand each other in, I think that’s more than enough.”
No preference	4	4	“I probably would not care if my significant other did not want to learn or does not speak Polish.”

Another prominent theme was *respect* (*n* = 13). Some participants expressed that what was most important was that their partner respected the fact that they would communicate in other languages with specific people (i.e., family members, target language community). For example:

“It is really important to have people in your life that are very respectful of the languages that you speak. I just want to add that respect is really reciprocal, right? If they are going to be respectful about how you’re speaking, how your family is speaking whatever languages they speak, you kind of give that back. That’s very important and a non-negotiable for me.”

However, some participants (*n* = 13) said that they would prefer it if their partner was more actively engaged in their family, and open to learning their HL so that they could easily communicate with family members. One participant said:

“It would obviously be nice if someone who’s important to me could communicate with my family to a certain degree. They don’t have to be fluent by any means. But it would be nice to be able to hold regular conversations, just because translating isn’t that fun.”

The last question had the purpose of summarizing all of their ideas and experiences discussed earlier in the focus group interview. We inquired, keeping in mind everything we had talked about (opportunities, challenges, changes in language use) if they were interested in retaining their childhood FLPs in their future families. A total of 38 responses were given, with the majority of participants (*n* = 22) commenting that not only would they be interested in retaining their FLPs, but they were also open to incorporating additional languages into their policies. One possible explanation for this addition would be if their partner spoke an additional language, that then would be integrated into their FLP. For example, one participant stated:

“I feel like I’m open to change. It’s not like I just want my language to be spoken, but my future wife’s as well. I’m just open to any changes. If there are any. But I would like my language as well to be incorporated into that change.”

A total of seven participants conveyed a need to simply retain their childhood FLPs and improve the languages they already speak. For example:

“I’d like to learn new words and add to my vocabulary in the future. I really just see myself trying to improve both the languages and any of the language policies I have now. I guess I would just like to better them and better myself.”

Lastly, only three participants reported that they do not plan to retain their HL. One participant stated: “I have thought about that. But I know that I myself cannot really speak Hindi and Gujarati that well, so I think I would probably struggle trying to teach it to my children on top of that.”

It is important to note that policy retention is not linked to one policy type. The following is a quote from a participant who was exposed to an implicit policy and stressed how ineffective it was in creating a solid foundation for their HL:

“I would essentially update it to perhaps be a bit more strict about it than my parents were. I essentially would copy their mannerisms and learn from that. We did have books in Russian but my parents didn’t have that much time to teach me properly in a way.”

This notion of implicit family language policies being ineffective is more consistent with older literature. For example, in Kasuya’s (1998) study where parents encouraged their children to speak more Japanese through the implementation of several different discourse strategies, it was concluded that “overall, the explicit strategy had the highest success rate in relation to the child’s subsequent choice of Japanese” (Kasuya, 1998, p. 342).

Conversely, explicit policies during childhood could also be perceived as counterproductive for intergenerational retention:

“So again, that kind of felt forced, which is probably why the language didn’t stick as well. […] I find it quite difficult to imagine that I’ll continue with the Ukrainian language, and it’s quite sad. But that’s just how I found myself growing up; it’s just been heavily English-focused.”

This idea of explicit policies being not the most effective resonates with Lo Bianco’s (2010) observation that “[o]vert, explicit and formal language policies that support multilingualism will not, on their own, achieve intergenerational language retention […] if the social, cultural, economic and political messages of a society promote linguistic uniformity” (Lo Bianco, 2010, p. 58).

Although some participants felt language policies, implicit or explicit, had little bearing on intergenerational transmission, others felt they were important. Some people emphasized that explicit childhood FLPs were effective in terms of (future) generational policy retention. For example:

“My parents very heavily encouraged us to speak in Urdu so that we remember the language. […] When I have kids in the future, I would also definitely want to pass down Urdu to them. I think it’s super important to keep a language alive. And I hope to teach them that.”

Others felt that intergenerational transmission was a matter of course, and did depend on implicit policy:

“For me, it’s more of a natural thing. I don’t really have to think about what language I’m going to speak. […] I also want to pass down Arabic, since it’s an integral part of my culture and my religion. If I hadn’t known Arabic, I feel like it would be very difficult for me to be Muslim.”

In sum, external factors such as implicit versus explicit policy exposure are not accurate predictors of generational policy retention. More accurate predictors of this retention appeared to be internal factors such as a person’s motivation, priorities, and connection with whom they can practice their HLs (Table 9).

**Table 9:** Summary of themes for the question “Are you interested in retaining your language policies?”.

Theme	Number of responses	Number of participants	Example
Integrate	25	22	“I’d be open to learning new languages.”	Improve existing languages	8	7	“I definitely would like to get better at the languages that I already speak first.”
Subtract	5	3	“I find it quite difficult to imagine that I’ll continue with the Ukrainian language.”

## Discussion

The main objectives of this study were to examine multilingual emerging adult Canadians’ experiences of family and school language policies in their childhood, and how these experiences might be linked to their decisions to retain their HL and their imagined FLP in their future family. In the following, we discuss the findings relating to these objectives, and then, along with a consideration of some of the study’s limitations, we suggest some directions for future research.

### Childhood family and school policies

When asked about their childhood experience with language policies, no predominant type of policy emerged; half reported they experienced implicit and a third indicated they experienced explicit language policies in the home. Moreover, among those who were exposed to an explicit FLP, the policies varied in their focus on the HL and/or the SL, depending on parents’ beliefs about the functional value of a language. Explicit policies were not unmalleable; several people indicated that explicit FLPs became less restrictive and more implicit over time due to geographical, social, and developmental reasons. Participants who moved to Canada at a younger age (under 12) experienced less of a dramatic shift in the enforcement of their FLPs compared to those who arrived at a later age. In terms of participants born in Canada, whether the FLP was implicitly or explicitly enforced, their policies had more stability over time. Overall, these findings suggest that young adults may have more or less clearly articulated childhood FLPs to draw on in considering their future family’s FLP; whether they endorse or resist their childhood FLP, a more explicit FLP might be more useful for tailoring an FLP for one’s future family.

In addition to FLPs, school language policies also influenced language retention and intentions for the future. In line with Ballinger et al. (2022), Sandel (2003), and Slavkov (2017), language policies enforced at school influenced subsequent home-speaking practices. Some participants indicated that their FLPs changed due to educational reasons such as starting school, changing schools, or changing language programs in school. Almost all participants were exposed through their education to languages other than the HL and SL, particularly French, the minority official language in the province in which the study took place. This high rate of involvement in French education is consistent with recent information from Statistics Canada (2024b) which indicates that almost half of French immersion students in Canada come from immigrant families. Although almost half of the respondents indicated that they chose to take a language course for personal interest, a similar proportion enrolled primarily due to program requirements. Given the importance of meaningful, personally endorsed choices for language learning and maintenance (Comanaru and Noels, 2009; Landry et al., 2022), future research might explore whether additional languages learned through compulsory education are later retained and integrated into future FLPS to the same extent as languages learned under more voluntary circumstances.

### The multilingual experience

Multilinguals face many challenges including social judgment, restricted opportunities, and experiences of language anxiety. Despite these downsides, most (but not all) participants were interested in retaining their HL and transmitting it to their offspring, mainly so that they could retain a connection with their HL community and culture, but also for a variety of pragmatic reasons. These plans were complicated by insecurities about language skills and restricted opportunities for interaction. Although concerns around social judgment from HL and SL speakers were mentioned, most participants declined to label these experiences as blatantly discriminatory. Most participants looked to these multilingual experiences, both positive and negative, to shape their future FLPs, meaning that, while both were influential, these current experiences probably had more of a direct impact on future FLPs compared to childhood FLPs.

### HL retention and FLPs in one’s future family

The relationship participants have with their childhood language policies is crucial for their future FLP. There was no clear association between the explicitness of a policy and the intention to transmit the language to the next generation. The participants who were interested in retaining and adding additional languages to their FLPs expressed how they desired to use the language(s) for their own benefit or enjoyment and not due to community, family, or personal pressures. Some participants mentioned the interconnection between home language and culture retention and were thus motivated to retain their HL for cultural and religious reasons. Moreover, most were also open to adding more languages to the FLPs in their future homes. The addition of languages to FLPs was associated with changes in geography or finding a partner that speaks additional languages. For both of these hypothetical situations, most participants were open to the integration of both partners’ language(s) into an FLPs (i.e., combining two childhood language policies into one home). This potential complexity of future FLPs could reasonably be expected, given the hyperdiversity of Canada and the local municipal region, as well as the social norms and ideologies favoring multiculturalism and bi- and multilingualism.

### Limitations and future directions

Our study provides insight into the attitudes of emerging adults’ future language use and language policy retention. However, some methodological limitations ought to be addressed in future research. First, since this is a retrospective study, the childhood memories that participants recalled may be selective or incomplete. However, given that participants’ remembered experiences (rather than their actual experiences) may inform their current and future intentions, the findings are nonetheless informative, as they reveal what the participants regarded as important childhood memories about their language use. Second, focus groups are limited in terms of the depth of description available for each participant’s experiences, and they should be complemented by individual interviews. With that said, we must highlight that focus group interviews allowed the participants to elaborate on the complexity of their language experiences, and to resonate (or not) with others’ observations, something that could not have been as effectively accomplished through other methods.

Despite these limitations, the current findings provide a map for other avenues of exploration. For instance, the diversity of experiences articulated in this small sample of focus group interviews should be followed using methods that are better suited to surveying a larger and broader sample, with attention given to the factors (e.g., personal, network, and societal factors and dynamics; Landry et al., 2022) that differentiate patterns of intentions across subgroups. Given the dynamic nature of FLPs, future research might consider a longitudinal design to gain a more nuanced understanding of how FLPs evolve as children develop and contexts change. Moreover, although young, single adults’ attitudes toward language retention are informative, it would certainly also be important to examine couples’ intentions, particularly with the birth of the first and later children. Researchers also might compare specific language groups, and whether and how these groups value and support transgenerational language use depending on their ethnolinguistic context, including language ideologies, opportunities for language use, and the tenor of relations between ethnolinguistic groups.

It is also important to note concerns about the generalizability of the results. Because we sampled a highly educated population, the results on the positive attitudes toward bilingualism/multilingualism may not be generalized to other contexts where an individual’s bilingualism is not acknowledged or appreciated. A possible future direction for researchers would be to focus on the multilingualism of Indigenous languages prominent in Canada.

## Conclusion

The retention of HLs is a necessary step to ensuring the maintenance of diverse languages within a society, a resource that offers many societal benefits. Focusing on multilingual Canadian emerging adults, their generational language retention is more complex than solely examining the interplay between an HL and an SL. While we have found no direct association between the explicitness of language policies at home and generational HL retention, additional factors must be taken into an adult’s intention to retain a language. These factors include school language policies, personal motivations, a future spouse’s values on language use, changes in geography and family connections, as well as balancing the opportunities and challenges of continuing to speak a language. As this study shows, a critical part of this research agenda is to ask the next generation about their intentions in this matter. Future research should continue to examine strategies that support language retention in increasingly multilingual societies.

## Data Availability

The raw data supporting the conclusions of this article will be made available by the authors, without undue reservation.

## Footnotes

^1^As employed in the present article, “home language” is in many respects similar to “heritage language” (particularly as it is used in the Canadian context; Nagy, 2021). It should be noted that with adult participants, it is possible that the home language of the participants’ current residence may not be the same as their home language when living with their family of origin. Moreover, the current home language of the family of origin may have changed since the participants’ childhood. For the reasons described by Eisenchlas and Schalley (2020), we chose the term “home language” while recognizing the complexities of this terminology.
