# Supplementary material for: Family language policy retention across generations: childhood language policies, multilingualism experiences, and future language policies in multilingual emerging Canadian adults
Source: Front Psychol. 2024 Sep 10;15:1394027. doi: 10.3389/fpsyg.2024.1394027 (PMC11422779; doi:10.3389/fpsyg.2024.1394027)
Supplement: Supplementary file 1 [file Data_Sheet_1.docx]

**Appendix A: Languages Spoken by Participants**

|  | Number of speakers |
| --- | --- |
| Amharic | 1 |
| Arabic | 8 |
| Bangla | 2 |
| Cantonese | 1 |
| Cebuano Bisaya | 1 |
| Farsi | 2 |
| French | 8 |
| Gujarati | 1 |
| Hindi | 13 |
| Hungarian | 1 |
| Italian | 2 |
| Korean | 2 |
| Mandarin | 3 |
| Marathi | 1 |
| Marwadi | 1 |
| Mongolian | 1 |
| Pashto | 1 |
| Polish | 1 |
| Punjabi | 8 |
| Romanian | 1 |
| Russian | 1 |
| Sinhalese | 2 |
| Somali | 1 |
| Spanish | 5 |
| Tagalog | 4 |
| Tigrinya | 1 |
| Ukrainian | 1 |
| Urdu | 11 |
| Vietnamese | 1 |

**Appendix B: Interview Questions**

Introductions:

1. Your name, pronouns, and age
2. What languages do you speak fluently?

Childhood linguistic questions:

1. Describe your family’s linguistic background.
   1. Which languages do you speak to which family member?
   2. How do you respond to each person?
2. What about at school?
   1. What languages did you learn/speak at school? (This includes primary school to university)
   2. Looking back, would you have made this choice for yourself? Do you believe your schooling was effective?
3. Which language(s) are you most comfortable speaking in social settings besides school (with different friend groups, at the store… )?
4. Has your language use changed over time (which language you speak to which person, in which context)?
   1. Were there any events in your life that caused this change? (e.g., moving, switching schools…)
5. In any of these situations, were you ever explicitly told which language(s) you had to speak?
   1. Or was it more natural, like you were getting clues from the languages other people were speaking?

Bilingual/multilingual identity questions:

1. What were some opportunities you faced because you can speak more than one language?
2. What were some of the challenges you faced?
   1. Did you have any discriminatory experiences?
   2. What would you consider to be disrespectful?

Language anxiety questions:

1. Have you ever experienced anxiety when speaking one of your languages?
2. If so, with whom? (ex: parents, grandparents, native speakers…)
3. What do you think caused this anxiety? (ex: afraid of making mistakes or being judged…)
   1. How do native speakers try to encourage you/discourage you? (e.g., correcting you, ignoring your mistakes…)

Family planning questions

1. How important is it for your significant other or your close friends to speak all the languages you speak?
   1. How important is it for them to understand all the languages you speak?
   2. What about simply respecting the fact that you communicate in other languages?
   3. Do they need to put in some effort to learn these languages?
2. How important is it that they can communicate with your family members who do not speak the same language they do?
3. Have you thought about which language(s) you will address your future children?
   1. Will you be explicit in telling them which languages they can speak and when?
   2. Or will this be more organic?
4. What about school?
   1. Which languages will they learn in school?
5. How important is it for you to retain these language policies that you were exposed to in your own home?
   1. Do you believe you will?
   2. Or will these policies be appropriately adjusted? (e.g., being exposed to new languages in the family…)
